# The Impact of COVID-19 on Medical Education: A Lost Generation of Ophthalmologists?

**DOI:** 10.7759/cureus.51790

**Published:** 2024-01-07

**Authors:** Janvi Karia, Ishika Bansal, Swapnil Parmar

**Affiliations:** 1 Division of Medicine, University College London, London, GBR

**Keywords:** hybrid learning, ophthalmology, undergraduate, education, covid-19

## Abstract

Purpose: COVID-19 had a significant impact on undergraduate medical education. There has been extensive analysis on the generic impact on medical education, but the individual impact on specialties, particularly ophthalmology, has not been widely researched. We explored the impact of COVID-19 on the undergraduate ophthalmology experience in the UK, characterising the effect on medical education and considered positive outcomes that could be implemented in future undergraduate curricula.

Methods: An online-cross sectional study was performed using a 13-item questionnaire in 2023, completed by 101 participants from UK medical schools. This study was conducted through University College London, England.

Results: Statistical and qualitative analysis revealed a significant reduction in clinical exposure during the pandemic with an almost complete shift to online lecture-based teaching. This teaching style has been adapted post-pandemic, which is impacting student confidence in dealing with ophthalmic conditions and deterring them from considering ophthalmology as a career.

Conclusions: COVID-19 has exacerbated a pre-existing gap in training medical trainees to deal with ophthalmic presentations. With an increase in the proportion of undergraduate medical education taking place online, efforts should be made to ensure students have hands-on, clinical exposure especially in practical placements, such as ophthalmology.

## Introduction

The COVID-19 pandemic significantly impacted all aspects of life and healthcare, including medical education. The first national lockdown in the UK was announced in March 2020 [1], social distancing rules were enforced, and the focus of healthcare shifted to acutely manage the repercussions of this contagious virus. Medical educators had to adopt a hybrid teaching style (mixture of in-person and online) or exclusive virtual teaching, differing from traditional methods.

Ophthalmology is a postgraduate surgical speciality. UK medical students have clinical teaching and placements in ophthalmology to encourage interest in pursuing the speciality and to expose students to presentations they may see in future practice. Currently, there is little research in the literature analysing the impact of COVID-19 on undergraduate ophthalmology and students' experiences. 

The specific objectives of this research were therefore to firstly explore students' perceptions on the impact of COVID-19 on their undergraduate teaching experience in ophthalmology. A secondary aim was to consider whether this had affected student interest in pursuing the speciality.

## Materials and methods

This study was conducted through University College London, England. An online-cross sectional study was performed using a questionnaire created with Google Forms (see Appendix). The questionnaire collected quantitative and qualitative data. There was a total of 13 questions (Likert-type, multiple choice, and free text) across three main topics (see Table 1).

**Table 1:** Information collected in the questionnaire

Topic	Information gathered
Demographics	Completion of clinical ophthalmology rotation or not, place of study, time period of rotation
Details of placement	Length of placement; formats of learning, e.g., online lectures, attending theatre sessions; students’ feedback on experiences missed during placement
Impact of COVID-19	Students’ views on whether their placement was impacted by COVID-19; perceptions of future impact on care; students’ desire to pursue the speciality in the future based on their experience during their placement

The questionnaire was distributed to medical students across the UK. There were 101 participants including medical students who had completed their ophthalmology placements and junior doctors who had qualified in the UK. Prior to data collection, three medical students from different medical schools and clinical years were invited to pilot the questionnaire to ensure relevance and consistency of the questions. The questionnaire was refined using feedback from these students, which largely consisted of improving clarity of the questions. 

Undergraduate medical students and recently qualified doctors were invited to complete the questionnaire between January and April 2023. It was advertised via departmental posters and university medical society mailing lists. The exclusion criteria are as follows: (1) participants who did not undergo undergraduate medical training the UK, (2) participants who did not complete a clinical ophthalmology rotation, and (3) participants who completed their clinical ophthalmology rotation over five years ago.

Microsoft Excel (Microsoft Corporation, USA) and IBM SPSS Statistics for Windows (IBM Corp., Armonk, New York, United States) were used to conduct descriptive and quantitative analysis. Statistical significance level was set to 5% and statistical tools used to compare relationships between categorical variables. P values <0.05 were considered statistically significant. Qualitative data were analysed independently by three researchers.

This study was approved by the University College London Research Ethics Committee (reference 22543/001). A participant information sheet was provided at the start of the questionnaire, which participants were asked to read. This explained their role in the study and asked them to proceed with the questionnaire if they were happy to provide informed consent. No identifiable data were collected, and questionnaires were completed voluntarily and anonymously.

## Results

Cohort demographics

The questionnaire was completed by 101 participants. Of this, 13 participants had not completed an ophthalmology rotation and therefore could not complete the questionnaire. One participant did not complete the entire questionnaire, and therefore their data were excluded. This resulted in a total of 87 valid responses (n = 87). The participants were from 18 different universities across the UK. Of note, one individual who had not had an ophthalmology placement further explained that their placement was cancelled due to the COVID-19 pandemic, but this had not been rescheduled.

To analyse how the teaching experience may have differed during COVID-19, responses were grouped into three groups: ‘pre-COVID’, ‘peak COVID’ and ‘post COVID’. In the UK, the first national lockdown was announced in March 2020, and the last of the COVID restrictions were lifted in December 2021 [1]. Therefore, responses from the participants whose ophthalmology rotation occurred prior to March 2020 were categorised into the ‘pre-COVID’ group for data analysis (n = 17). Participants with placements between March 2020 and December 2021 are grouped into ‘peak COVID’ (n = 37), and those who had their placement after December 2021 are categorised as ‘post COVID’ (n = 33). This structured approach to data analysis allowed for a systematic evaluation of results. 

Impact of COVID-19 on ophthalmology placement

**Figure 1:**
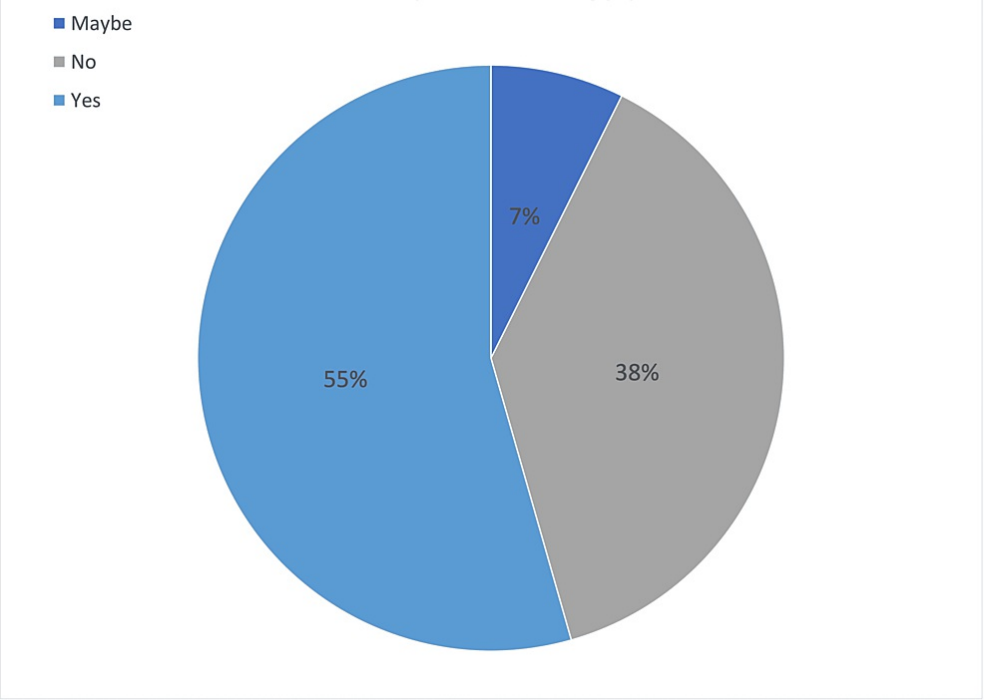
Proportion of participants reporting an impact of COVID-19 on their ophthalmology placement after March 2020

The participants were asked if they felt that the pandemic impacted their undergraduate ophthalmology placement. Most participants (55%) from both the peak and post-COVID categories combined felt that their placement was affected by COVID-19, as seen in Figure 1.

**Figure 2:**
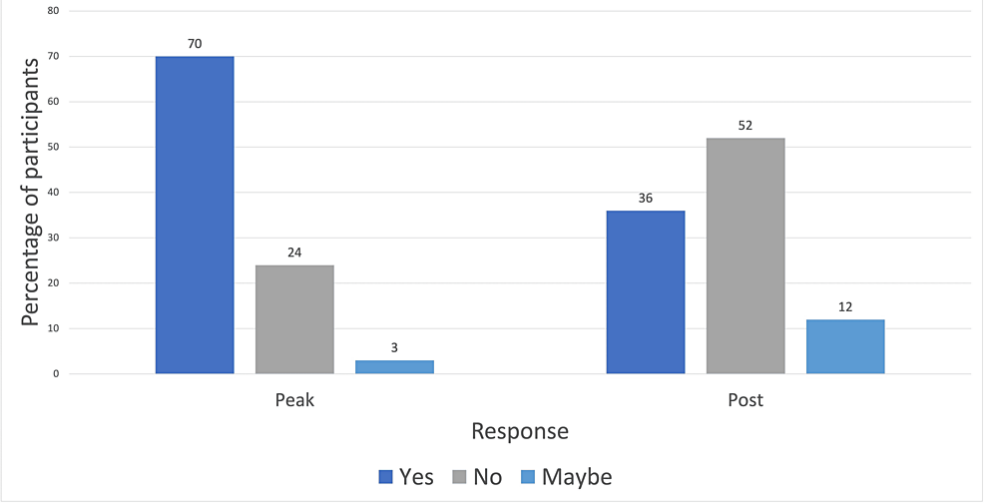
Impact of COVID-19 in those with placements during peak COVID compared to post-COVID

This was analysed further to differentiate between those in the peak and post-COVID groups, as presented in Figure 2.

Chi-squared test was performed, and a statistically significant difference was found between the peak and post-COVID groups: x^2^(2, n = 60) = 26.2, critical value: 5.99, p = <0.01. 

Reasons for perceived impact

There were a variety of reasons that the participants found that their placements were impacted. Qualitative responses were examined and are categorised in Table 2. 

**Table 2:** Reasons reported by the participants for the impact on their COVID-19 ophthalmology placements.

Reasons for perceived impact	Number of participants
Reduced or no in-person clinics and theatre sessions	29
Lack of staffing	2
Shortened placement	5
Social distancing - for example, limitation on number of people allowed in clinic room	2
Telephone consultations/clinics - for example, reduced ability to examine patients face to face	1
Limited experience in specialist centres	4

Format of teaching

The participants were asked what type of teaching sessions they had, including online and in-person teaching sessions/lectures, clinics and theatres. In the pre-COVID cohort, all participants had some form of in-person teaching. In the peak COVID group, 56% had exclusively online teaching, while 44% had some form of in-person teaching. Post-COVID, no participants described exclusive online teaching. A large proportion had a hybrid form of teaching (combination of both online and in-person teaching). Only 15% had exclusive in-person teaching in this post COVID group.

Interest in pursuing the speciality

The participants were asked to rate on a scale from 1 to 5 their interest in pursuing ophthalmology as a speciality based on their placement, with 5 being most likely. To evaluate if a relationship exists between the type of teaching received during the placement and the interest in pursuing the speciality, the following were considered: 1) Type of exposure manually coded to fall into either 'online' or 'in-person', whereby participants selected being exposed to any form of in-person activity (in addition to any online exposure), i.e., in-person theatres, in-person clinics or in-person seminars. This was coded as 'in-person'. The participants who had exclusive online sessions are coded as 'online'. 2) The participants selected a value from 1 to 5 in response to the following question 'My ophthalmology placement in medical school made me more likely to pursue ophthalmology in the future'. The values corresponded as 1 for strongly disagree, 2 for disagree, 3 for neutral, 4 for agree and 5 for strongly agree. 

The assumption that less than 20% of cells have an expect count of less than 5 was not met for this dataset, and therefore the P-value for the likelihood ratio is used, P = 0.082. This suggests that there is no significance between the type of exposure received during the undergraduate ophthalmology rotation and the likelihood of pursuing this speciality. 

The above data were supported by qualitative responses given by the participants for why they are more or less likely to pursue the speciality. Responses were categorised into experiences that made them more likely or less likely to pursue the speciality. Specific quotations are included in Table 3 to illustrate the points.

**Table 3:** Reasons given for why the participants were more or less likely to pursue ophthalmology after the placement.

Reasons given for why participants were more or less likely to pursue ophthalmology after placement			
More likely		Less likely	
‘Placement was interesting and varied’	1	Insufficient exposure: ‘Since this was not a proper exposure to ophthalmology, it was difficult to tell’. ‘No experience so I have no idea what the speciality is truly like and whether I want to pursue it’.	19
‘Good overview of training process’	1	Felt like it was a competitive environment.	3
‘Enjoyed being involved in theatre time’	2	Short placement: ‘I was interested in it at the time but did not spend enough time there during my rotation to have a substantial shift in my specialty preferences.’	3
‘Early exposure helped gauge whether it was suitable for me’	1	Practical limitations due to COVID-19 rules: ‘Could not examine patients, could not use slit lamps’. ‘Poor staffing so everything was cancelled’.	3
‘Good exposure to what the speciality is about’. Further reasons provided included: Good work-life balance and friendly teams really impact patients' lives.	6	Reasons related to ophthalmology observed from their placement: ‘Skills out of reach’, ‘Operating field too small’, ‘Wasn’t able to see anything apart from cataracts', ‘The glaucoma clinic was very boring’, ‘The one clinic I went to was boring’.	5

Thirty-three percent of the responses found that the placement did not affect their decision-making as they already knew that they ‘weren’t drawn to the speciality’ or ‘did not have any interest in the speciality before or after placement'. Four individuals within this group found that the placement is enjoyable and has ‘good overview’ and ‘good exposure’, but it did not change their decision-making.

Impact on patient care

**Figure 3:**
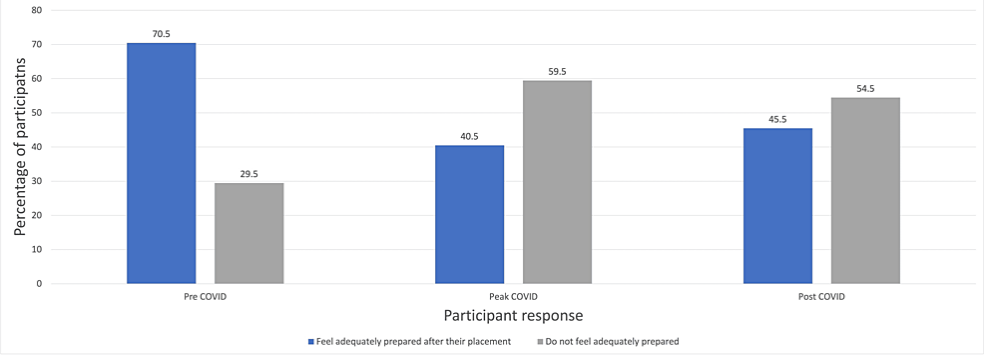
Graph showing if the participants felt adequately prepared to treat ophthalmic conditions after their ophthalmology placement

The foremost role of medical school placements is to prepare medical students for their practice as doctors. Ophthalmic presentations could occur in a range of specialties, and doctors should have sufficient knowledge and skill to review patients. This could ensure more detailed referrals to ophthalmology, allowing them to prioritise patient that need to be reviewed more urgently. The participants were asked if any reduction in their ophthalmology placement led to a reduction in their confidence in treating patients with ophthalmic problems. As Figure 3 shows, there was less confidence in the participant groups affected by COVID-19.

The participants discussed the benefits of having in-person clinical exposure to allow them to examine patients and manage emergency presentations, with multiple participants (5) remarking that their knowledge came solely from independent bookwork. Some participants were able to encounter ophthalmic conditions in settings, such as GP and ED (emergency department).

## Discussion

Impact on ophthalmology teaching

To consider the impact on ophthalmology teaching, it is useful to firstly consider the effects of COVID-19 on ophthalmology services in the UK. Ting et al. [2] evaluated the effects of COVID-19 on ophthalmology service provision in the UK. There was over 60% reduction in both outpatient and surgical activities, which has caused a large backlog in the system, somewhat mitigated by an increase in virtual consultations [2]. The Royal College of Ophthalmology also described redeployment of trainees and reduction of trainee opportunities, such as simulation and staff illness [3].

This reduction in in-person clinical activity correlates with both the quantitative and qualitative responses of the questionnaire. Seventy percent of all participants with placements after March 2020 felt that their placement was affected by COVID-19. The most reported reason for this was reduced or no in-person clinical exposure, such as clinics and theatre sessions. Other reasons cited were lack of staffing and social distancing. These are factors that influenced medical schools to cancel clinical placements. Ferrel et al. reported that due to COVID-19 restrictions and change of clinical prioritisation to focus on the treatment of COVID-19 patients, 'in-person teaching and clinical clerkships were cancelled'[4].

To mitigate the loss of in-person opportunities, many universities shifted in-person placements to online teaching, which has been noted throughout the literature. As per Dost et al. [5], students reported an increase in time spent using online teaching platforms from four to six hours prior to the pandemic to seven to 10 hours per week.

Format of teaching

This questionnaire examined the format of teaching. According to participant responses, there was a shift from no online teaching prior to the COVID-19 pandemic to some participants receiving exclusively online teaching during the peak of the pandemic. In the post-COVID cohort, a majority of participants continued to describe a hybrid version. There was a statistically significant difference in the proportion of students reporting an impact in the peak COVID group compared to the post-COVID group (X2 (2 degrees of freedom, n = 66) = 24, p = 0.01), correlating with a larger proportion of online teaching in this group.

The shift to online teaching was generally noted to be negative in our research, with 58% of participants reporting ‘insufficient exposure to the speciality'. This has been explored by Ferrel et al. who noted a loss in ability to collaborate and gain 'the real time feedback and back and forth that develop in class that are hard to replicate in online forums' [4]. The loss of social connection can increase feelings of isolation [6].

One of the main barriers to online teaching noted [5] was the difficulty in teaching clinical skills online. In a surgical speciality, such as ophthalmology, competency in examining patients and with instruments, such as slit lamps and ophthalmoscopes, is difficult to teach online. A study in the University of Utah trialling an online format for clinical electives found online teaching of ophthalmology to be effective, but ‘extensive teaching’ students lacked confidence in performing examinations' [7]. Other logistical factors that impact the efficacy of online methods include Internet connection and availability and external distractions, such as family at home and timing of tutorials [5].

On the other hand, online teaching can enable a greater level of independence and flexibility. Online teaching can provide flexibility in allowing students to access it from anywhere. If it is a recorded material, they can pause and revisit later [8]. A systematic review considering learning methods found that ‘blended’ or mixed online and in-person teaching methods showed a consistent benefit when compared to traditional learning [9]. Contributing factors included the use of multimedia to enrich learning, such as videos and audio. Additionally, studies have explored the impact of innovative new technologies, such as virtual reality and other immersive technologies, to supplement teaching [10].

The post-COVID cohort continued to report a ‘hybrid’ model of teaching, and as discussed above, there are significant benefits. However, going forward, educators should consider how to mitigate the negative impacts that students face, such as by including in-person clinical skills and considering ways to improve feedback mechanisms that maximise connection between teachers and students.

Confidence in dealing with ophthalmic presentation

Undergraduate ophthalmology teaching is an essential aspect of the curriculum regardless of whether they wish to pursue the speciality in the future. For those interested in a future career in ophthalmology, the teaching experience allows students to explore what training can be like, allow themselves to be involved in research and enhance their portfolio. For those not interested, this can be the only exposure to the develop the necessary skills to tackle ophthalmic presentations. Many systemic diseases have ocular manifestations and can often be the first presentation of these conditions (e.g., peripheral corneal ulcers in rheumatoid arthritis) [11]. Consequently, these acute eye problems are often seen in GP (5% of all consultations) [12] and A&E (around 1.46-6% of attendance) [13], making it essential for future doctors to be prepared regardless of future specialty.

This study highlighted that a higher proportion of students with an ophthalmology rotation in peak (61%) and post COVID (52%) felt that they were not adequately prepared to treat ophthalmic conditions in comparison to pre-COVID (33%). The reasons students often cited for their perceived negative impact on patient care in the future was due to 'lack of clinical exposure to ophthalmic patients' and 'little practical experience with examination and fundoscopy'. Research taken place pre-COVID already show that there is low confidence among different specialty doctors in dealing with eye problems. A survey carried out among doctors in the A&E department by Murray, Benjamin and Oyede found that 57% of doctors felt that their undergraduate teaching was inadequate and a high proportion of doctors are not confident in using ophthalmic equipment, like ophthalmoscopes (71%) and slit lamp (68%) [14]. Zhang et al. also assessed confidence among family medicine doctors and found that 80% of residents are 'somewhat comfortable' or 'not at all comfortable' in assessing and managing eye problems [15]. Both studies highlight that prior to COVID-19, there was already a gap in training medical students in dealing with ophthalmic issues. During peak/post-COVID, this gap in knowledge and low confidence had likely worsened. Harvey et al. surveyed 1145 medical students and highlighted that a high proportion of medical students doubted their competence with medical knowledge and skills due to the lack of clinical exposure, cancellation of end-of-year assessments and no clinical skills teaching during their medical school experience in the pandemic [16]. Some of these themes were also highlighted within the qualitative responses of this study. The COVID-19 pandemic has had a negative impact on the confidence of medical students to manage ophthalmic presentations in the future due to changes with their experiences in medical school. The likely effects will need to be further studied to see whether there is an increase in the misdiagnosis and mismanagement of common eye problems or an increase in ophthalmology referrals from primary care or ED.

Interest within the specialty

There has been some research into what influences medical students during their undergraduate training to pursue a specific specialty. Many differing factors have been identified, but the most common ones include clinical mentor [17], personal interest [18] and perceptions of specialty characteristics [19]. It is likely that the shift from in-person to online teaching will influence these factors as the lack of exposure could make it more difficult for students to explore ophthalmology as a specialty and gain a clinical mentor to help develop further interest. Lo et al. surveyed medical students regarding ophthalmology residency applications specifically and found COVID-19 to have had a negative impact on students applying. Reasons cited were lack of elective placements in ophthalmology and reduction in clinical opportunities and research [20].

In this study, the results did show that those students who were less likely to pursue ophthalmology in the future mentioned that the lack of clinical experience within the field and COVID-19 restrictions preventing practical skills as reasons why. However, overall, there was no significant relationship between the type of the teaching students received (in-person vs. online) and their decision to pursue ophthalmology in the future (p = 0.082). Possible explanations for these results are that clinical exposure may not have a big influence on medical students' interest to pursue that specialty as there are other ways for them to build and explore careers. For example, there has been an increased use in social media, which has not only served use in educational purposes but also a means of possible communication and collaboration with senior clinicians [21] who can act as clinical mentors and allow medical students to understand the breadth of a particular specialty.

Limitations and further work

Data were gathered based on the perceptions of impact, but students will not have had their placements both prior to the COVID-19 pandemic and after it had started; therefore, the comparison may be biased. In addition, long-term effects could be compared with objective measures, such as by evaluating the number of referrals from primary care or competition ratios for entry to ophthalmology speciality training. A further limitation is that the questionnaire did not utilise a binary 'positive' or 'negative' to specifically differentiate the nature of the impact. Instead, the analysis of perceived positive and negative was based on the researcher's interpretation of the short answer responses.

## Conclusions

Medical schools have had to adopt a new style of delivering teaching to students during the COVID-19 pandemic. There was a complete shift from the traditional in-person teaching to online teaching. Whilst this novel method allows increased accessibility to education for students, some disadvantages identified include reduction in both clinical exposure to patients and refining practical skills. These essential aspects of education are needed to develop confidence and interest within specialties like ophthalmology.

Since COVID-19 restrictions have ended, many universities have integrated these new methods into their curriculum to form a hybrid style of teaching. This has the potential to bridge these disadvantages and allow for a more flexible learning environment for students. However, it remains to be seen what the long-term impact of this change will be in ophthalmology.

## Appendices

**Table 4:** Questionnaire distributed to the participants

Ophthalmology teaching in COVID-19 This is a questionnaire designed to understand the impact of the COVID-19 pandemic on undergraduate ophthalmology teaching in the UK. This questionnaire is designed for students who have completed or are undertaking their ophthalmology rotation and doctors who have finished medical school. By completing the questionnaire, you provide consent for us to use your answers for our project. All data are kept secure and remain anonymous. Please ensure you do not provide any personal data when answering the questions. Please open the following link to view the Participant Information Sheet, which provides more information about this project: https://tinyurl.com/yrxksy8v. Thank you so much for your time!	
Have you completed your clinical ophthalmology rotation in medical school? (Yes or No)	
Which medical school do you/did you study at?*	
Have you had exposure to ophthalmology in any of the following formats while at medical school? (tick all that apply)	Online lectures In person lectures; In-person clinics; In-person theatres; Online teaching sessions/seminars In person sessions/seminars; Other
How long was your placement?*	1 week or less; Between 1 and 2 weeks; More than 2 weeks
In which period of time did your ophthalmology rotation happen?*	2023; July 2022-Dec 2021; Jan-June 2021; April-Dec 2020; Jan-March 2020; 2019-Before 2019; Other
Do you feel that your placement was impacted by COVID-19?* Please provide an explanation for your answer above*	Yes; No; Maybe
What experiences during your ophthalmology rotation (if any) did you miss out on? e.g. theatres, clinics, simulation sessions	
If you missed some ophthalmology teaching or experience, do you think it will impact on care for your patients with ophthalmic problems? Please explain your answer to the question above.	Yes; No
Was your placement affected more, less or the same as other speciality placements in the same clinical year?	More; Less; The same
My ophthalmology placement in medical school made me more likely to pursue ophthalmology in the future. Please explain your answer to the question above.	Rate from 1-5 (1 is strongly disagree, 5 is strongly agree)
Submission: Please ensure you have not provided any personal data such as your name in your answers to the questions before submitting the form.	
